# Baseline Predictors of Visual Acuity Outcome in Patients with Wet Age-Related Macular Degeneration

**DOI:** 10.1155/2018/9640131

**Published:** 2018-02-26

**Authors:** Xinyuan Zhang, Timothy Y. Y. Lai

**Affiliations:** ^1^Beijing Institute of Ophthalmology, Beijing Tongren Eye Center, Tongren Hospital, Capital Medical University, Beijing 100730, China; ^2^Pao So Kok Macular Disease Treatment and Research Centre and Department of Ophthalmology & Visual Sciences, No. 4/F, Hong Kong Eye Hospital, The Chinese University of Hong Kong, 147K Argyle Street, Kowloon, Hong Kong; ^3^2010 Retina and Macula Centre, Kowloon, Hong Kong

## Abstract

Age-related macular degeneration (AMD) is one of the leading causes of severe vision loss in people over 60 years. Wet AMD (wAMD) causes more severe visual acuity (VA) loss compared with the dry form due to formation of choroidal neovascularization (CNV). Antivascular endothelial growth factor (anti-VEGF) agents such as ranibizumab and aflibercept are now the standard of care treatment for wAMD. Unfortunately, up to a quarter of anti-VEGF-treated wAMD patients might not fully benefit from intravitreal injections and CNV activity may not respond to the treatment and these patients are called anti-VEGF nonresponders. This article aims to discuss the baseline factors associated with VA outcome such as age, initial VA, lesion types, disease duration, optical coherence tomography (OCT) features, fundus autofluorescence findings, and the presence of particular genotype risk alleles in patients with wAMD. Recommendations are provided regarding when to consider discontinuation of therapy because of either success or futility. Understanding the predictive factors associated with VA outcome and treatment frequency response to anti-VEGF therapy may help retina specialists to manage patients' expectations and guide treatment decisions from the beginning of treatment on the basis of “personalized medicine.”

## 1. Introduction

Over the past two decades, eight anti-VEGF drugs have been approved by US Food and Drug Administration (US FDA). Among these, three are commonly used for the treatment of wet age-related macular degeneration (wAMD), which is the leading cause of blindness in people aged over 60 years. Pegaptanib (Macugen, Eyetech Pharmaceuticals/Pfizer) was the first VEGF inhibitor approved by the USFDA for treating wAMD in December 2004. It is a selective anti-VEGF RNA aptamer to VEGF-165 but it is no longer widely used due to inferior effectiveness compared to other anti-VEGF agents. In February 2004, VEGF inhibitor bevacizumab (Avastin, Genentech, San Francisco) was approved for the first-line treatment of metastatic colorectal cancer and it also became a commonly used off-label drug for wAMD [1]. Another agent, ranibizumab (Lucentis, Genentech/Novartis) received FDA approval in 2006 for the treatment of wAMD [2]. Another anti-VEGF agent, aflibercept (Eylea, Regeneron/Bayer), was approved by the US FDA in November 2011. It is a fusion protein known as VEGF trap which binds to VEGF-A, VEGF-B isoforms, and placental growth factor (PlGF). The most recent anti-VEGF drug is conbercept (Chengdu Kanghong pharmaceuticals, Chengdu, China), which is another VEGF trap similar to aflibercept. It was approved for the treatment of wAMD by China FDA in December 2013.

Although anti-VEGF therapy has been a breakthrough in the treatment of wAMD, unfortunately, up to a quarter of anti-VEGF-treated wAMD patients might not benefit from intravitreal injections and choroidal neovascularization (CNV) activity does not respond to the treatment. Understanding the predictive factors associated with visual acuity outcome and treatment response to anti-VEGF therapy may help retina specialists to manage patients' expectations and guide treatment decisions at treatment initiation. In this review, we discuss various definitions of responder, poor responder, delayed responder, and nonresponder. Preclinical data illustrating the mechanisms of adaptive evasion to anti-VEGF therapy will also be summarized. Finally, we will emphasize the possible predictors to VA improvement and how to achieve the best VA outcome after anti-VEGF therapy for wAMD in clinical practice.

## 2. Responder, Poor Responder, Delayed Responder, and Nonresponder

A nonresponder is originally defined as a person or animal who does not show any immune response following vaccination against a specific virus. Self-reported tumor resistance to anti-VEGF treatment or so-called nonresponders appears more frequently. An ocular nonresponder to anti-VEGF treatment refers to those patients who developed reduced distance or reading VA compared to baseline during follow-up. These patients can be called “initial nonresponder,” “recalcitrant' wAMD,” or “tachyphylaxis.” Lux et al. reported that 45% of the patients with wAMD are nonresponders who underwent intravitreal injections of 1.25 mg bevacizumab and were followed up for 6 months. The nonresponders were defined as follows in this study: (1) reduction in both visual acuity and reading ability at the last follow-up; (2) reduction in either visual acuity or reading ability at the last follow-up; (3) no change in either visual acuity or reading ability at the last follow-up [1]. It has been shown that 14.3% of polypoidal choroidal vasculopathy (PCV) and 14.3% of eyes with wAMD required additional photodynamic therapy (PDT) treatment due to lack of response to intravitreal ranibizumab treatment [2]. The VISION study reported that 40% of eyes with wAMD treated with pegaptanib lost at least 15 letters from the baseline [3]. In the MARINA study, only 34.5% in the 0.3 mg ranibizumab and 42.1% in the 0.5 mg ranibizumab group had better than 20/60 visual acuity [4]. In the ANCHOR study, nonresponder accounted for 10.1% in 0.5 mg ranibizumab-treated eyes which lost at least 15 letters at month of 24 [5]. Nagai et al. [6] defined patients who had no improvement of best corrected visual acuity (BCVA) and lack of reduction of OCT central retinal thickness (CRT) at the end of the initial treatment as initial nonresponders. According to the definition, baseline VA and macular morphology are the two important parameters to assess the treatment response following anti-VEGF therapies. Amoaku et al. proposed the followings as good response to anti-VEGF therapy among wAMD patients: resolution of intraretinal fluid (IRF), subretinal fluid (SRF), and retinal thickening, and/or improvement of at least 5 ETDRS letters. If there was <25% reduction in OCT CRT from the baseline value, with persistent or new IRF, SRF, or minimal or no change in VA (VA change of 0 to 4 letters) after VEGF therapy, it is defined as poor response. Nonresponder could be defined as having increase in fluid (IRF, SRF, and CRT), or increase in hemorrhage compared with the baseline and/or loss of >5 letters compared with the baseline BCVA [7].

## 3. Polypoidal Choroidal Vasculopathy (PCV) Is More Common among Nonresponders to Anti-VEGF Therapy

Polypoidal choroidal vasculopathy (PCV), characterized by the choroidal vascular abnormalities, was first described by Yannuzzi et al. in 1982. The prevalence varies among different ethnic groups and Asian carries the highest prevalence with up to 53% [8], as compared with the prevalence of around 10% in Caucasian populations [9]. wAMD and PCV share some common clinical features and genetic risk factors. However, the pathological process, natural history, response to anti-VEGF therapy, and treatment outcomes might be quite different.

It has been reported that nearly 50% of nonresponders are misdiagnosed for typical wAMD and PCV accounted for the majority of these cases. As shown by several retrospective observational case series, PCV was the main reason (80–90%) of resistance to anti-VEGF treatment in patients with wAMD [10–12].

The mechanism of the different response to anti-VEGF drugs in eyes with PCV and wAMD is still not fully understood. Pathological studies have revealed that fibrosis and proliferation of retinal pigment epithelium (RPE) cells are more prominent features in wAMD than in PCV and the pathogenesis of polypoidal lesions or the branching vascular network may not purely depend on increased levels of VEGF [13–15]. It has been found that the aqueous humors level of VEGF is significantly lower in eyes with PCV than those of wAMD (*P* = 0.045); furthermore, levels of PDGF significantly increased in eyes with PCV, wAMD, and pathological myopia, suggesting that the role of VEGF in the pathogenesis of wAMD is greater than in the pathogenesis of PCV [13]. The differential expression level between in patients with PCV or wAMD can at least partially explain why PCV is the majority nonresponder among wAMD patients.

However, a number of studies recently suggested that the response to anti-VEGF therapy in PCV patients may differ according to anti-VEGF drugs. In a retrospective, interventional case series, the effectiveness of intravitreal injection of aflibercept and ranibizumab for patients with PCV was compared. After a 12-month follow-up, there was no significant difference in BCVA between the two groups; however, aflibercept more often led to polyp regression than ranibizumab [16]. Kawashima et al. also found that patients with wAMD or PCV patients refractory to ranibizumab, switching to aflibercept might be more effective regardless of patients' genotype [17]. Further study with large sample size is warranted to compare the efficiency of different drugs in PCV patients.

## 4. Mechanisms of Nonresponse to Anti-VEGF Therapy

The mechanism of nonresponse to anti-VEGF therapy is still poorly understood despite being previously studied intensively in cancer research. After the approval of bevacizumab by the USFDA for various cancers treatment including glioblastoma (GBM), it has been demonstrated that bevacizumab improved the radiographic response, progression-free survival, and quality of life of patients with GBM. However, there are still a proportion of patients who developed resistance and failed to respond to anti-VEGF therapy. Mechanisms postulated to be associated with lack of response to anti-VEGF therapy include (1) activation of the by-pass angiogenesis pathway through upregulation of other proangiogenic factors such as basic fibroblast growth factor (bFGF), stromal cell-derived factor-1 alpha (SDF-1alpha), and platelet-derived growth factor-C (PDGF-C); (2) recruitment of bone marrow-derived stem cells (BMDSC) concomitant with increased level of proangiogenic cytokines and chemokines in the endothelial progenitor (EPC); (3) pericytes progenitors cells (PPC) invading normal tissue areas; and (4) autocrine effects of VEGF signaling promoting tumor invasiveness [14].

Other proposed mechanisms for resistance to anti-VEGF therapy of a tumor cell may be due to the activation of c-Met gene. VEGF receptor (VEGFR) is complexed with c-Met effectors. Hypoxia-induced increase expression of HGF or c-met and prompt ligand independent activation of c-Met. Activation of c-Met gene subsequently induces tumor cell inactivation and transformation [18]. The expression of proangiogenic factors such as angiogenin, IL-1alpha, IL-1beta, TNF-alpha and TGF-alpha, matrix metalloproteinase- (MMP-) 2, MMP-9, and MMP-12 secreted protein acidic and rich in cysteine (SPARC); and tissue inhibitor of metalloproteinase (TIMP) 1 was elevated at mRNA and protein level after anti-VEGF therapy in U87 and NSC23 cell lines and it was found that bFGF was correlated with reactive of tumor angiogenesis [19].

A number of studies have also elucidated the mechanisms of nonresponsiveness following anti-VEGF therapy in ocular diseases. Macrophages induced by anti-VEGF therapy may accelerate the tolerance of anti-VEGF therapy. This may explain the fact that the increased intravitreal dosage of anti-VEGF agents may not contribute and increase the treatment response. Moreover, capillary stabilization in adults is a VEGF independent process. The vascular wall of capillary is surrounded by a single layer of pericytes which stabilizes the vessels during its development and this process is VEGF independent [20]. Finally, the process of angiogenesis is complex and involves multiple molecular and cellular transduction pathways. It has been suggested that VEGF-A is just one of the main pathogenic factors involved in wAMD. PIGF and platelet-derived growth factor (PDGF) have also been shown to be involved in the development of CNV. Animal model has suggested that PIGF, a homologous factor to VEGF, is not essential for physiological angiogenesis but is an important regulator in the pathological angiogenic conditions [21]. Many studies have demonstrated that pericytes share a common basement membrane with endothelial cells and can produce survival factors which shield endothelial cells from anti-VEGF therapy [22–24]. Anti-PDGF has been shown to inhibit angiogenesis in both human and animal studies [25]. Phase IIb trial also demonstrated favorable safety and efficacy profiles of PDGF (pegpleranib) and anti-VEGF drug combination therapy for wAMD across multiple clinically relevant end points [26]. However, the recently released outcomes of two phase III clinical trials using either pegpleranib (Fovista) or rinucumab both showed no additional benefit in adding anti-PDGF agents when compared with anti-VEGF monotherapy. Further research in the role of anti-PDGF in wAMD is warranted.

Therefore, anti-VEGF therapy alone may play only a partial role in the inhibition of CNV and anti-VEGF agents used in combination with drugs affecting other angiogenesis mechanisms may yield better results. For instance, clinical and laboratory studies suggested that dual inhibition of VEGF and PDGF may be more effective than targeting VEGF alone [25]. Further research is warranted to study the possible role of the elevated level of some particular proteins in the vitreous such as cytokines, chemokines, and other molecular regulators to explain the mechanisms of the resistance to anti-VEGF therapy.

## 5. Baseline Predictors of Visual Acuity Outcome in wAMD

### 5.1. Patients Characteristics

The baseline visual acuity (VA) is one of the most important predictor for final VA outcomes as it will provide the floor or ceiling effect. Patients with worse VA might be correlated with better VA improvement after treatment and patients with better VA are less likely to gain as much due to ceiling effects. In a subgroup analysis of MARINA and ANCHOR study, if the baseline VA in one group is higher than another group by 5 letters, the mean change of VA from baseline to 24 months will be lower by 3.2 letters in the better initial VA group [27]. Vitreoretinal adhesions have also been found to be significantly correlated with poor clinical outcome. Baseline CNV lesion size has been found in several studies to be associated with VA outcomes and large CNV area generally corresponds to poorer visual acuity outcome [27–30]. Several studies have shown that younger age is correlated with better clinical outcomes. In the subgroup analysis of MARINA study, if the average age of one group is younger than another group by 13.7 years at baseline, the change in VA of the younger group will be 5 letters better than the older group [27]. Similarly, subgroup analysis of ANCHOR also showed that younger patients gained more compared with the older group [30]. CATT study also found that patients less than 70 years old gained 10.8 letters, while patients 70 years or older only gained 5.6 letters after treatment [28]. The interval between onset of symptoms and commencement of treatment is another important baseline predictor for final visual outcome and shorter interval from presentation to treatment is correlated with better VA outcomes. It has been shown that patients with a delay in treatment of 21 weeks or more compared to a delay of 7 weeks or less had an odds ratio of 2.62 for worsening vision after treatment, suggesting that longer delay of treatment of commencement was a significant predictor of poorer treatment outcome [29].

### 5.2. Parameters of Optical Coherence Tomography (Table 1)

Optical coherence tomography (OCT) allows noninvasive high resolution imaging of the retina in vivo [31, 32]. Integrity of ellipsoid zone (IS/OS) is highly correlated with VA clinical outcome. Absence or disruption of this layer has been demonstrated to be abnormality of photoreceptor or choroidal diseases [33–38]. It has been found that ellipsoid zone is disrupted in 55%–65% of patients with advanced AMD. In a hospital-based study, eyes with wAMD received intravitreal anti-VEGF treatment. The final visit VA is closely correlated with the integrity of VA. The prognosis VA of patients who have a complete ellipsoid zone is better than those which have partially complete or the invisible ellipsoid zone. Interruption of IS/OS layer is correlated with poor visual prognosis and the length of ellipsoid zone disruption is correlated with different VA outcome [34]. Integrity of external limiting membrane (ELM) is also directly correlated with the VA [35]. Interrupted ellipsoid zone is a sign of the destruction of outer segment of photoreceptor; however, interruption of ELM is a sign of serious damage of inner segment or cell bodies of photoreceptor. In a one-year follow-up of aflibercept treatment on wAMD, the status of ELM is good predictor for visual outcome of wAMD and VA [36, 37]. It has been shown that the integrities of ellipsoid zone and ELM are both correlated with better final VA. Eyes with completely intact ellipsoid layer but disrupted ELM generally have poorer VA (worse than 20/200), indicating that ELM might be a more reliable predictor of VA than ellipsoid zone [38].

It remains controversial whether there is any correlation between foveal retinal thickness (FRT) and VA outcome. It is well accepted that FRT is an early sign and sensitive parameter for detecting reduced baseline VA; however, it is not correlated with the VA outcome [39]. In a hospital-based study with a total of 1105 subjects, in all treatment groups, age, larger CNV area, and greater foveal thickness are negatively correlated with VA outcome [29]. However, another study has shown that central retinal thickness (CRT) is not correlated with VA outcome but is an early sensitive predictor of decreased VA [39]. Patients with intraretinal fluid (IRF) and RPE high reflectivity have been shown to have poor VA outcome [39]. In a 12-month follow-up study, it has been demonstrated that, in eyes that were treated with 3 loading doses of ranibizumab or bevacizumab followed by as needed injections, the mean BCVA was significantly better in eyes with no IRF compared with eyes which had persistent IRF (*P* < 0.05), while the visual improvement in BCVA was similar between eyes with or without SRF eye [40]. It has been shown that IRF may increase the risk of the formation of geographic atrophy. The 2-year outcome from the CATT study has shown that eyes with IRF under fovea had twice the chance of developing GA and increased subretinal fluid and sub-RPE tissue thickness were associated with a decreased risk for development GA [41]. Results from the subgroup analysis of HARBOR study also showed that, at the 3-, 12-, and 24-month follow-up, SRF is the protective factor for the formation of geographic atrophy compared with the eyes without SRF (2% versus 10%, 5% versus 24%; 8% versus 33%v, respectively) in patients with wAMD [42].

Baseline choroidal thickness might be another important OCT for VA prognosis as it is well accepted that abnormalities of choroidal vasculature are involved in the pathogenesis of wAMD [43]. Age, axial length, refractive error, blood pressure, intraocular pressure, and diurnal variation are influent factors with the thickness of choroid [44]. As there is a relative lack of data from the normal subjects in cohort population study, normal choroid thickness (CT) also varies depending on the method used, and the number of subjects enrolled. In a small case serial study, CT is measured by enhanced depth imaging (EDI) OCT from the posterior border of the retinal pigment epithelium to the choroid/sclera junction at 500 *μ*m to 2500 *μ*m temporal and nasal to the fovea and central 1-mm area of the choroid. The mean central macular thickness was 216.4 ± 30.03 *μ*m and choroid was found to be the thinnest nasally and thickest subfoveally. On multivariate regression, age was the most significant factor affecting subfoveal CT (*P* < 0.001). Regression analysis showed an approximate decrease in CT of 1.18 *μ*m every year [45]. Manjunath reported that the CT is thinnest nasally, thickest in the subfoveal region, and thicker temporally, with the mean subfoveal CT of 272 *μ*m (SD, ±81 *μ*m) [46]. Increased choroidal thickness has been shown to be closely associated with wAMD [47, 48]. Spectral domain (SD) OCT and swept source OCT provide more accurate information of choroidal thickness by using EDI mode of the imaging software [49]. It has been shown that significant reduction of choroidal thickness is correlated with improved VA after intravitreal treatment of ranibizumab in wAMD patients [50]. Baseline CT is also regarded as a predictive factor of VA outcome in patients with wAMD. In a retrospective, consecutive case series study, greater baseline subfoveal choroidal thickness was found to be associated with a better anatomic and functional clinical outcome in eyes with wAMD after intravitreal aflibercept treatment (*r* = 0.98, *P* < 0.0001) [51]. Subfoveal choroidal thickness is also a predictor of macular GA development [52].

Another OCT feature to assess in wAMD eyes is the double layer sign, which is one of the OCT features of PCV indicating abnormal choroidal BVN associated with PED. The dual highly reflective layers can be identified by OCT, one at the level of the RPE and another beneath the RPE. ICGA examination is recommended to rule out PCV in these cases and these cases might potentially be less responsive to anti-VEGF therapy [53].

### 5.3. Fluorescein Angiography (FA) and Indocyanine Green (ICGA) Findings

FA is useful to document the size of CNV and it has been shown that smaller size of CNV is correlated with good visual prognosis. The MARINA trial showed that if the CNV size in group B is bigger than group A by 3.6 disc area (DA), at the end of study, the VA in group B is lower than group A by 5 letters. ANCHOR study which followed patients for 12 months showed that the lesions increased 1 DA; VA is lower by about 3.54 letters. Furthermore, it was also shown that larger size of CNV is correlated with higher proportion of eyes with complete disruption of the Ellipsoid Zone [30]. FA and ICGA can also evaluate the subtypes of wAMD and these subtypes include classic CBV, predominately classic CNV, occult CNV, PCV, and retinal angiomatous proliferation (RAP) [54]. Multiple studies have shown that CNV subtypes are correlated with the VA outcomes. In a hospital-based study, 106 patients who received intravitreal anti-VEGF treatment, type I neovascularization at baseline, were more likely to maintain good vision over 4 years [31]. Kang and Roh reported that CNV size not CNV type is correlated with patient's VA outcome [55]. It has also been found that eyes with occult CNV and RAP significantly increase in VA after 3 injections compared to eyes with occult CNV without RAP (*P* < 0.01). No other differences were observed between CNV lesion types regarding VA or change in VA [56].

### 5.4. Vitreomacular Interface Abnormalities

Vitreomacular interface abnormality (VMIA) in patients with wAMD includes vitreomacular adhesion (VMA), vitreomacular traction (VMT), and epiretinal membrane (ERM). These have been found to correlate with nonresponder to anti-VEGF therapy. It has been found that VMA is more common in eyes with wAMD as compared to control eyes with nonvascular AMD [57–59]. 12.8% of the 1185 patients in the CATT study were found to have VMT or VMA. Progression to GA occurred at a lower rate in eyes with VMT and VMA at baseline (11.7%) compared to eyes without VMT or VMA. On the other hand, a greater number of anti-VEGF injections was required in eyes with VMT or VMA over 2 years, suggesting that the presence of VMA and VMT at baseline is a predictor for nonresponse or tachyphylaxis to anti-VEGF therapy. The localization of VMA or VMT over CNV may hinder the penetration of anti-VEGF agents into the macula. This localization of VMA over CNV also suggests that inflammatory cytokines may participate in the pathogenesis of both CNV and VMA/VMT [53].

### 5.5. Outer Retinal Tabulation (ORT)

Outer retinal tabulation (ORT) is a tubular structure found in the outer retina which can be detected by OCT. In CATT study, the prevalence of ORT was 10.1% at 56 weeks and 17.4% at 104 weeks. The presence of ORT represents degeneration of photoreceptor cell and dysfunction of retinal epithelium cells and mitochondria. It also represents the rebuilding of inner segment of photoreceptors [60]. ORT detected by OCT is correlated with the histological distinguishable structure changes. Schaal et al. found that the location, the composition, and shape of ORT are closely correlated with histological changes (correspondence to four phases of cone degeneration), suggesting that presence of ORT is an indicator of cone degeneration and poor VA outcome [61]. It was shown that intravitreal injection of ranibizumab stabilized ORT and inhibited occurrence of ORT [62] which was a predictor of VA in eyes with center involved diabetic macular edema [63] and wAMD [64]. ORT is also a predictor of the enlargement of geographic atrophy in AMD [65].

### 5.6. Fibrovascular and Serous Pigment Epithelium Detachment (PED)

Fibrous tissue beneath the RPE may block the diffusion of oxygen and other nutrients from the choroidal layer to the retina and also affects the drug penetration from the vitreous to retina and choroid [66, 67]. Suzuki et al. found that fibrovascular PED (OR 33.5, 95% CI 2.95 to 381) is significantly associated with nonresponse to anti-VEGF therapy as judged by both BCVA and fundus findings [66].

### 5.7. Fundus Autofluorescence (FAF)

Detection of fundus autofluorescence is a noninvasive tool which has the potential to predict progression of AMD. Lipofuscin is the fluorophores visualized as by blue light (wavelength 488 nm) autofluorescence. Accumulation of lipofuscin has been shown to be correlated with aging and progression of wAMD [68]. Imagine detection of FAF together with OCT and FA are routine investigations performed in clinical trials of wAMD. FAF image provides useful information reflecting RPE functions. Although FAF is more widely used for dry AMD, it is generally accepted that an intact normal foveal FAF is a good predictor for response to anti-VEGF therapy [69]. Better VA improvement is correlated with less abnormality in FAF. Increased FAF indicates excessive accumulation of lipofuscin in RPE and poorer visual prognosis. Reduced FAF may suggest apoptosis of RPE and dysfunction [70].

## 6. Predictive or Pharmacodynamics and Biomarkers

Drug-related biomarkers (drug metabolizing enzymes, transporters and targets, etc.) and genetic polymorphisms have been evaluated as factors influencing drug effectiveness or individual differences in drug response. Successful completion of Human Genome Project is a strong impetus to the expansion of clinical medicine from the macro to micro areas and from cell to molecular level. In recent years, many studies have shown that biomarkers provide a good prognosis for the patient's individualized treatment, indicating that research has been taken into the molecular diagnosis of wet AMD and individualized treatment era.

## 7. Gene Variant

Genetic variants are potentially promising predictors for prognosis after anti-VEGF therapy for AMD. It has been found that a higher frequency of the risk (T) allele (Allelic *P* = 0.019) and TT genotype (*P* = 0.002 under a recessive model) for the* VEGFA*-rs943080 polymorphism are correlated with nonresponse to anti-VEGF therapy. VEGFA expression was 1.8-fold higher in cells with the* VEGFA *rs943080 TT genotype than in cells with the VEGFA rs943080 CC genotype (*P* = 0.012) [71]. Orilin et al. reported that single-nucleotide polymorphism rs1061170, rs10490924, rs3750848, rs3793917, rs11200638, and rs932275 and for the indel del443ins54 spanning the* CFH*,* ARMS2*, and* HTRA1 *genes are correlated with negative response after anti-VEGF therapy [72]. It was also found that individuals with genotype CC of p.Y402H in* CFH* had less chance of positive treatment outcome compared with those with the CT and TT genotypes (*P* = 0.005 and *P* = 0.006). In this study, the genotype combination of AG at* CFH *with CT at* FZD4 *(SNP rs10898563) was found to have an increased chance of positive treatment outcome (*P* = 0.004) [73]. Another study suggested that polymorphism rs1061170 in the* CFH *gene is a predictor of treatment response to anti-VEGF drugs [72].

## 8. Management Strategies for Nonresponders to Anti-VEGF Therapy Combination Therapy

Multiple studies have shown that combination therapy, administered in dual or triple combinations (corticosteroids, verteporfin photodynamic therapy, and anti-VEGF agents), might have more advantages compared with anti-VEGF monotherapy, especially in terms of reducing the need for retreatment. This is especially important for the PCV subtype of wAMD as the influence of VEGF appears to be lower in PCV. Another rationale of performing combination therapy is the potential increased expression of VEGF in PCV patients following PDT and anti-VEGF therapy which can counteract this post-PDT increase in VEGF production [14]. FOCUS study is the first clinical trial to evaluate the efficiency of combination anti-VEGF therapy with PDT in patients with wAMD. The result showed that, at month 24, 88% ranibizumab combined with PDT therapy patients lost <15 ETDRS letters from baseline VA compared with the PDT treatment alone and had low rate of adverse event [74]. Similarity, the MONT BLANC study reported that intraretinal cysts or SRF decreased significantly more in the combination group than the monotherapy anti-VEGF alone. Intraretinal cysts were the only relevant prognostic parameter for functional outcome [75]. EVEREST II study showed that VA improved by 8.3 letters in the combination therapy group compared with the 5.1 letters of the ranibizumab monotherapy group. The complete regression rate of polyps is also significantly higher than in the combination group compared with the ranibizumab monotherapy group, suggesting that initially combined therapy can be considered as the first-line treatment strategy for PCV [76]. In the DENALI study, it was shown that ranibizumab monotherapy or combination with PDT improved VA at 12 month, furthermore noninferiority (7-letter margin) of combination regimens to ranibizumab monotherapy was not shown [77]. In the PLANET study for PCV, it was demonstrated that aflibercept monotherapy with sham rescue PDT was noninferior to aflibercept combined with active rescue PDT in terms of visual acuity gain over 2 years. At week 52, both treatment arms gained over 10 letters from baseline, and the visual acuity gain was maintained until week 96. CST reduction from baseline was similar between the two treatment arms and polyps showed no activity in over 80% of patients. Nonetheless, PLANET study required fixed dosing of aflibercept with initial 3 loading doses at monthly interval followed by 2 monthly injections during the first year and it was unclear whether as needed treatment with aflibercept could achieve similar results.

## 9. Switch to Different Anti-VEGF Agents

Switching of anti-VEGF drugs can be considered in nonresponders following treatment of wAMD [69]. Ehlken et al. reported that nonresponders may benefit from switching to other drugs either to bevacizumab or ranibizumab. In this study, VA at the time of the switching, anti-VEGF therapy was the only prognostic factor for the progress of VA and positively correlated with the beneficial improvement of VA by linear regression analysis [69]. VA at the time of the switch was positively correlated with a beneficial development of VA after changing the drug. In addition, significant anatomical and visual benefits could be in nonresponders when switching from bevacizumab to ranibizumab [70]. Lucio-Eterovic et al. suggested that switching nonresponders to aflibercept may be a good option after failed ranibizumab or bevacizumab therapy [18]. Further research in a large population is warranted.

## 10. Summary

In conclusion, age, baseline vision, OCT features, and genetic polymorphisms at baseline might be potential prognostic predictors for VA in patients with wAMD. Genetic factors might be the causes for the variations in drug reactions among different individuals and races. With improvements of genomic technology platforms, better correlations of genotypic and phenotypic findings can be identified and this will allow better use of pharmacogenomics in individualizing therapy.

Innovation in the biochemical field has led to substantial clinical progress. The development and availability of new drugs and biological products will allow novel treatment options for patients, especially for those nonresponders to anti-VEGF therapy. Currently, there are several new drugs which underwent the preclinical, Phase I–III investigations: abicipar pegol, a recombinant protein of the designed Ankyrin repeat protein (DARPin, Allergan) family, is an antagonist of VEGF-A that inhibits all relevant subtypes of VEGF-A with high potency. In the phase 2b PEACH study for wAMD, it was reported that abicipar pegol provided at least equal or higher vision gains with the potential for fewer injections in compared to the standard of care treatment ranibizumab. Brolucizumab (ESBA1008, ALCON) was shown by OSPREY phase II study to have the similar effects with aflibercept. Inhibition of angiopoietin 2 involved in the transmembrane tyrosine kinase protein Tie2 pathway has been shown to reduce vascular leakage and inhibit angiogenesis in mouse model of wet AMD. A phase 1 trial which evaluated the drug RG7716 (Roche), a bispecific monoclonal antibody to VEGF and angiopoietin 2, has demonstrated good safety with positivity biologic signals in terms of both VA and anatomical improvements in patients wet AMD [78]. Furthermore, more than 20 new drugs are currently under clinical investigations including X82 (Tyrogenex), GB 102 (Graybug), OHR-120 (Santen), and THR-317 (Thrombogenics) for the treatment of wAMD. Further basic medical research and the rapid development in the field of biotechnology will provide critical insight into the clinical applicability of new regimens for the treatment of wAMD.

## Figures and Tables

**Table 1:** Prognostic impact of OCT imaging in patients with wAMD.

Anatomical structure	Significant findings	Relevant to the clinical outcome
Ellipsoid zone (EZ)	Absence or disruption	Highly correlated to visual outcome
External limiting membrane (ELM)	Interruption	A sign of damage of inner segment of cell bodies of photoreceptors
Foveal retinal thickness (FRT)	Thicker than normal	Controversial
Features of retinal and RPE layers	Presence of intraretinal fluid	Poorer VA outcome Increase the risk of geographic atrophy
Baseline choroidal thickness (CT)	Thicker subfoveal choroidal thickness	Poorer VA outcome
Retinal pigment epithelium (RPE)	Double layer sign	A predictor of PCV, higher risk of nonresponse to anti-VEGF therapy

wAMD: wet age-related macular degeneration.

## Abbreviations

AMD: Age-related macular degeneration
bFGF: Basic fibroblast growth factor
cFDA: China FDA
CNV: Choroidal neovascularization
CRT: Central retinal thickness
ELM: External limiting membrane
ERM: Epiretinal membrane
FVPED type: Fibrovascular RPE detachment
GBM: Glioblastoma
IRF: Intraretinal fluid
nAMD: Neovascular AMD
OCT: Optical coherence tomography
ORT: Outer retinal tabulation
PDGF: Platelet-derived growth factor
PED: Pigment epithelium detachment
PIGF: Placental growth factor
RAP: Retinal angiomatous proliferation
RPE: Retinal pigment epithelium
SD OCT: Spectral domain OCT
VEGFR: VEGF receptor
VMA: Vitreomacular adhesion
VMIA: Vitreomacular interface abnormalities
VMT: Vitreomacular traction
wAMD: Wet AMD.

## References

[1] Lux A., Llacer H., Heussen F. M. A., Joussen A. M. (2007). Non-responders to bevacizumab (Avastin) therapy of choroidal neovascular lesions. *British Journal of Ophthalmology*.

[2] Yamashiro K., Tomita K., Tsujikawa A. (2012). Factors associated with the response of age-related macular degeneration to intravitreal ranibizumab treatment. *American Journal of Ophthalmology*.

[3] Gragoudas E. S., Adamis A. P., Cunningham E. T., Feinsod M., Guyer D. R. (2004). Pegaptanib for neovascular age-related macular degeneration. *The New England Journal of Medicine*.

[4] Rosenfeld P. J., Brown D. M., Heier J. S. (2006). Ranibizumab for neovascular age-related macular degeneration. *The New England Journal of Medicine*.

[5] Brown D. M., Michels M., Kaiser P. K., Heier J. S., Sy J. P., Ianchulev T. (2009). Ranibizumab versus verteporfin photodynamic therapy for neovascular age-related macular degeneration: two-year results of the ANCHOR study. *Ophthalmology*.

[6] Nagai N., Suzuki M., Uchida A. (2016). Non-responsiveness to intravitreal aflibercept treatment in neovascular age-related macular degeneration: Implications of serous pigment epithelial detachment. *Scientific Reports*.

[7] Amoaku W. M., Chakravarthy U., Gale R. (2015). Defining response to anti-VEGF therapies in neovascular AMD. *Eye (Basingstoke)*.

[8] Kwok A. K. H., Lai T. Y. Y., Chan C. W. N., Neoh E.-L., Lam D. S. C. (2002). Polypoidal choroidal vasculopathy in Chinese patients. *British Journal of Ophthalmology*.

[9] Lafaut B. A., Leys A. M., Snyers B., Rasquin F., De Laey J. J. (2000). Polypoidal choroidal vasculopathy in Caucasians. *Graefe's Archive for Clinical and Experimental Ophthalmology*.

[10] Coppens G., Spielberg L. A., Leys A. (2011). Polypoidal choroidal vasculopathy, diagnosis and management. *Bulletin De La Societe Belge D'Ophtalmologie*.

[11] Gomi F., Tano Y. (2008). Polypoidal choroidal vasculopathy and treatments. *Current Opinion in Ophthalmology*.

[12] Tranos P., Vacalis A., Asteriadis S. (2013). Resistance to antivascular endothelial growth factor treatment in age-related macular degeneration. *Drug Design, Development and Therapy*.

[13] Tong J.-P., Chan W.-M., Liu D. T. L. (2006). Aqueous humor levels of vascular endothelial growth factor and pigment epithelium-derived factor in polypoidal choroidal vasculopathy and choroidal neovascularization. *American Journal of Ophthalmology*.

[14] Imamura Y., Engelbert M., Iida T., Freund K. B., Yannuzzi L. A. (2010). Polypoidal Choroidal Vasculopathy: A Review. *Survey of Ophthalmology*.

[15] Kumar Chhablani J. (2011). Photodynamic therapy for polypoidal choroidal vasculopathy. *Graefe's Archive for Clinical and Experimental Ophthalmology*.

[16] Cho H. J., Kim K. M., Kim H. S. (2016). Intravitreal Aflibercept and Ranibizumab Injections for Polypoidal Choroidal Vasculopathy. *American Journal of Ophthalmology*.

[17] Kawashima Y., Oishi A., Tsujikawa A. (2015). Effects of aflibercept for ranibizumab-resistant neovascular age-related macular degeneration and polypoidal choroidal vasculopathy. *Graefe's Archive for Clinical and Experimental Ophthalmology*.

[18] Lu K. V., Bergers G. (2013). Mechanisms of evasive resistance to anti-VEGF therapy in glioblastoma. *CNS Oncology*.

[19] Lucio-Eterovic A. K., Piao Y., De Groot J. F. (2009). Mediators of glioblastoma resistance and invasion during antivascular endothelial growth factor therapy. *Clinical Cancer Research*.

[20] Darland D. C., D'Amore P. A. (1999). Blood vessel maturation: Vascular development comes of age. *The Journal of Clinical Investigation*.

[21] Carmeliet P., Moons L., Luttun A. (2001). Synergism between vascular endothelial growth factor and placental growth factor contributes to angiogenesis and plasma extravasation in pathological conditions. *Nature Medicine*.

[22] Benjamin L. E., Hemo I., Keshet E. (1998). A plasticity window for blood vessel remodelling is defined by pericyte coverage of the preformed endothelial network and is regulated by PDGF-B and VEGF. *Development*.

[23] Bergers G., Hanahan D. (2008). Modes of resistance to anti-angiogenic therapy. *Nature Reviews Cancer*.

[24] Carmeliet P., Jain R. K. (2011). Principles and mechanisms of vessel normalization for cancer and other angiogenic diseases. *Nature Reviews Drug Discovery*.

[25] Kudelka M. R., Grossniklaus H. E., Mandell K. J. (2013). Emergence of dual VEGF and PDGF antagonists in the treatment of exudative age-related macular degeneration. *Expert Review of Ophthalmology*.

[26] Jaffe G. J., Ciulla T. A., Ciardella A. P. (2017). Dual Antagonism of PDGF and VEGF in Neovascular Age-Related Macular Degeneration: A Phase IIb, Multicenter, Randomized Controlled Trial. *Ophthalmology*.

[27] Boyer D. S., Antoszyk A. N., Awh C. C., Bhisitkul R. B., Shapiro H., Acharya N. R. (2007). Subgroup analysis of the MARINA study of ranibizumab in neovascular age-related macular degeneration. *Ophthalmology*.

[28] Lim J. H., Wickremasinghe S. S., Xie J. (2012). Delay to treatment and visual outcomes in patients treated with anti-vascular endothelial growth factor for age-related macular degeneration. *American Journal of Ophthalmology*.

[29] Ying G.-S., Huang J., Maguire M. G. (2013). Baseline predictors for one-year visual outcomes with ranibizumab or bevacizumab for neovascular age-related macular degeneration. *Ophthalmology*.

[30] Kaiser P. K., Brown D. M., Zhang K. (2007). Ranibizumab for predominantly classic neovascular age-related macular degeneration: subgroup analysis of first-year ANCHOR results. *American Journal of Ophthalmology*.

[31] Chae B., Jung J. J., Mrejen S. (2015). Baseline predictors for good versus poor visual outcomes in the treatment of neovascular age-related macular degeneration with intravitreal anti-VEGF therapy. *Investigative Ophthalmology & Visual Science*.

[32] Fercher A. F., Hitzenberger C. K., Drexler W., Kamp G., Sattmann H. (1993). In vivo optical coherence tomography. *American Journal of Ophthalmology*.

[33] Chang L. K., Koizumi H., Spaide R. F. (2008). Disruption of the photoreceptor inner segment-outer segment junction in eyes with macular holes. *Retina*.

[34] Mitamura Y., Mitamura-Aizawa S., Katome T. (2013). Photoreceptor impairment and restoration on optical coherence tomographic image. *Journal of Ophthalmology*.

[35] Coscas F., Coscas G., Lupidi M. (2015). Restoration of outer retinal layers after aflibercept therapy in exudative AMD: Prognostic value. *Investigative Ophthalmology & Visual Science*.

[36] Oishi A., Tsujikawa A., Yamashiro K. (2015). One-year result of aflibercept treatment on age-related macular degeneration and predictive factors for visual outcome. *American Journal of Ophthalmology*.

[37] Kwon Y. H., Lee D. K., Kim H. E., Kwon O. W. (2014). Predictive findings of visual outcome in spectral domain optical coherence tomography after ranibizumab treatment in age-related macular degeneration. *Korean Journal of Ophthalmology*.

[38] Shin H. J., Chung H., Kim H. C. (2011). Association between foveal microstructure and visual outcome in age-related macular degeneration. *Retina*.

[39] Gerding H., Loukopoulos V., Riese J., Hefner L., Timmermann M. (2011). Results of flexible ranibizumab treatment in age-related macular degeneration and search for parameters with impact on outcome. *Graefe's Archive for Clinical and Experimental Ophthalmology*.

[40] Wickremasinghe S. S., Sandhu S. S., Busija L., Lim J., Chauhan D. S., Guymer R. H. (2012). Predictors of AMD treatment response. *Ophthalmology*.

[41] Grunwald J. E., Daniel E., Huang J. (2014). Risk of Geographic Atrophy in the Comparison of Age-related Macular Degeneration Treatments Trials. *Ophthalmology*.

[42] Ho A. C., Busbee B. G., Regillo C. D. (2014). Twenty-four-month efficacy and safety of 0.5 mg or 2.0 mg ranibizumab in patients with subfoveal neovascular age-related macular degeneration. *Ophthalmology*.

[43] Koh L. H. L., Agrawal R., Khandelwal N., Sai Charan L., Chhablani J. (2017). Choroidal vascular changes in age-related macular degeneration. *Acta Ophthalmologica*.

[44] Tan C. S., Ouyang Y., Ruiz H., Sadda S. R. (2012). Diurnal variation of choroidal thickness in normal, healthy subjects measured by spectral domain optical coherence tomography. *Investigative Ophthalmology & Visual Science*.

[45] Chhablani J., Rao P. S., Venkata A. (2014). Choroidal thickness profi le in healthy Indian subjects. *Indian Journal of Ophthalmology*.

[46] Manjunath V., Taha M., Fujimoto J. G., Duker J. S. (2010). Choroidal thickness in normal eyes measured using cirrus HD optical coherence tomography. *American Journal of Ophthalmology*.

[47] Kim Y. T., Kang S. W., Bai K. H. (2011). Choroidal thickness in both eyes of patients with unilaterally active central serous chorioretinopathy. *Eye*.

[48] Manjunath V., Goren J., Fujimoto J. G., Duker J. S. (2011). Analysis of choroidal thickness in age-related macular degeneration using spectral-domain optical coherence tomography. *American Journal of Ophthalmology*.

[49] Chhablani J., Barteselli G. (2015). Clinical applications of choroidal imaging technologies. *Indian Journal of Ophthalmology*.

[50] Kang H. M., Kwon H. J., Yi J. H., Lee C. S., Lee S. C. (2014). Subfoveal choroidal thickness as a potential predictor of visual outcome and treatment response after intravitreal ranibizumab injections for typical exudative age-related macular degeneration. *American Journal of Ophthalmology*.

[51] Gharbiya M., Iannetti L., Parisi F., De Vico U., Mungo M. L., Marenco M. (2014). Visual and anatomical outcomes of intravitreal aflibercept for treatment-resistant neovascular age-related macular degeneration. *BioMed Research International*.

[52] Lee J. Y., Lee D. H., Lee J. Y., Yoon Y. H. (2013). Correlation between subfoveal choroidal thickness and the severity or progression of nonexudative age-related macular degeneration. *Investigative Ophthalmology & Visual Science*.

[53] Cuilla T. A., Ying G. S., Maguire M. G. (2015). Influence of the vitreomacular interface on treatment outcomes in the comparison of age-related macular degeneration treatments trials. *Ophthalmology*.

[54] Sebag J. (2015). Vitreous in age-related macular degeneration therapy - The medium is the message. *Retina*.

[55] Kang S., Roh Y.-J. (2009). One-year results of intravitreal ranibizumab for neovascular age-related macular degeneration and clinical responses of various subgroups. *Japanese Journal of Ophthalmology*.

[56] Ristau T., Hillebrand S., Smailhodzic D. (2013). Prognostic Factors for Long Term Visual Acuity Outcome after Ranibizumab Therapy in Patients with Neovascular Age-Related Macular Degeneration. *Clinical Experimental Ophthalmology*.

[57] Nomura Y., Takahashi H., Tan X., Fujimura S., Obata R., Yanagi Y. (2014). Effects of vitreomacular adhesion on ranibizumab treatment in Japanese patients with age-related macular degeneration. *Japanese Journal of Ophthalmology*.

[58] Lee S. J., Lee C. S., Koh H. J. (2009). Posterior vitreomacular adhesion and risk of exudative age-related macular degeneration: paired eye study. *American Journal of Ophthalmology*.

[59] Waldstein S. M., Sponer U., Simader C., Sacu S., Schmidt-Erfurth U. (2012). Influence of vitreomacular adhesion on the development of exudative age-related macular degeneration: 4-year results of a longitudinal study. *Retina*.

[60] Zweifel S. A., Engelbert M., Laud K., Margolis R., Spaide R. F., Freund K. B. (2009). Outer retinal tubulation a novel optical coherence tomography finding. *JAMA Ophtalmology*.

[61] Schaal K. B., Freund K. B., Litts K. M., Zhang Y., Messinger J. D., Curcio C. A. (2015). Outer retinal tubulation in advanced age-related macular degeneration optical coherence tomographic findings correspond to histology. *Retina*.

[62] Hua R., Liu L., Hu Y., Chen L. (2015). The occurrence and progression of outer retinal tubulation in Chinese patients after intravitreal injections of ranibizumab. *Scientific Reports*.

[63] Sun J. K., Lin M. M., Lammer J. (2014). Disorganization of the retinal inner layers as a predictor of visual acuity in eyes with center-involved diabetic macular edema. *JAMA Ophthalmology*.

[64] Faria-Correia F., Barros-Pereira R., Queirós-Mendanha L. (2013). Characterization of neovascular age-related macular degeneration patients with outer retinal tubulations. *Ophthalmologica*.

[65] Hariri A., Nittala M. G., Sadda S. R. (2015). Outer retinal tubulation as a predictor of the enlargement amount of geographic atrophy in age-related macular degeneration. *Ophthalmology*.

[66] Suzuki M., Nagai N., Izumi-Nagai K. (2014). Predictive factors for non-response to intravitreal ranibizumab treatment in age-related macular degeneration. *British Journal of Ophthalmology*.

[67] Friedlander M. (2007). Fibrosis and diseases of the eye. *The Journal of Clinical Investigation*.

[68] Brunk U. T., Terman A. (2002). Lipofuscin: mechanisms of age-related accumulation and influence on cell function. *Free Radical Biology & Medicine*.

[69] Ehlken C., Jungmann S., Böhringer D., Agostini H. T., Junker B., Pielen A. (2014). Switch of anti-VEGF agents is an option for nonresponders in the treatment of AMD. *Eye (Basingstoke)*.

[70] Heimes B., Lommatzsch A., Zeimer M. (2008). Foveal RPE autofluorescence as a prognostic factor for anti-VEGF therapy in exudative AMD. *Graefe's Archive for Clinical and Experimental Ophthalmology*.

[71] Saitta A., Nicolai M., Neri P., Reibaldi M., Giovannini A., Mariotti C. (2015). Rescue therapy with intravitreal aflibercept for choroidal neovascularization secondary to choroidal osteoma non-responder to intravitreal bevacizumab and ranibizumab. *International Ophthalmology*.

[72] Chen H., Yu K.-D., Xu G.-Z. (2012). Association between variant Y402H in age-related macular degeneration (AMD) susceptibility gene CFH and treatment response of AMD: A meta-analysis. *PLoS ONE*.

[73] Kloeckener-Gruissem B., Barthelmes D., Labs S. (2011). Genetic association with response to intravitreal ranibizumab in patients with neovascular AMD. *Investigative Ophthalmology & Visual Science*.

[74] Antoszyk A. N., Tuomi L., Chung C. Y., Singh A. (2008). Ranibizumab Combined With Verteporfin Photodynamic Therapy in Neovascular Age-related Macular Degeneration (FOCUS): Year 2 Results. *American Journal of Ophthalmology*.

[75] Ritter M., Simader C., Bolz M. (2014). Intraretinal cysts are the most relevant prognostic biomarker in neovascular age-related macular degeneration independent of the therapeutic strategy. *British Journal of Ophthalmology*.

[76] Koh A., Lee W. K., Chen L.-J. (2012). Everest study: Efficacy and safety of verteporfin photodynamic therapy in combination with ranibizumab or alone versus ranibizumab monotherapy in patients with symptomatic macular polypoidal choroidal vasculopathy. *Retina*.

[77] Larsen M., Schmidt-Erfurth U., Lanzetta P. (2012). Verteporfin plus ranibizumab for choroidal neovascularization in age-related macular degeneration: Twelve-month MONT BLANC study results. *Ophthalmology*.

[78] Campochiaro P. A., Khanani A., Singer M. (2016). Enhanced Benefit in Diabetic Macular Edema from AKB-9778 Tie2 Activation Combined with Vascular Endothelial Growth Factor Suppression. *Ophthalmology*.

